# Supplementary material for: Dynamic changes in somatosensory and cerebellar activity mediate temporal recalibration of self-touch
Source: Commun Biol. 2024 May 3;7:522. doi: 10.1038/s42003-024-06188-4 (PMC11068753; doi:10.1038/s42003-024-06188-4)
Supplement: Supplementary file 2 — Description of Additional Supplementary Files [file 42003_2024_6188_MOESM2_ESM.pdf]

Description of Additional Supplementary Files

File name: Supplementary Data 1

Description: The source (descriptive) data behind the graphs of Figure 4.
