# Supplementary material for: Dynamic changes in somatosensory and cerebellar activity mediate temporal recalibration of self-touch
Source: Commun Biol. 2024 May 3;7:522. doi: 10.1038/s42003-024-06188-4 (PMC11068753; doi:10.1038/s42003-024-06188-4)
Supplement: Supplementary file 4 — Reporting Summary [file 42003_2024_6188_MOESM4_ESM.pdf]

nature portfolio

Corresponding author(s): Konstantina Kilteni

Last updated by author(s): Apr 3, 2024

Reporting Summary

Nature Portfolio wishes to improve the reproducibility of the work that we publish. This form provides structure for consistency and transparency in reporting. For further information on Nature Portfolio policies, see our Editorial Policies and the Editorial Policy Checklist.

Statistics

For all statistical analyses, confirm that the following items are present in the figure legend, table legend, main text, or Methods section.

n/a	Confirmed	
☐	☒	The exact sample size (*n*) for each experimental group/condition, given as a discrete number and unit of measurement
☐	☒	A statement on whether measurements were taken from distinct samples or whether the same sample was measured repeatedly
☐	☒	The statistical test(s) used AND whether they are one- or two-sided<br>*Only common tests should be described solely by name; describe more complex techniques in the Methods section.*
☐	☒	A description of all covariates tested
☐	☒	A description of any assumptions or corrections, such as tests of normality and adjustment for multiple comparisons
☐	☒	A full description of the statistical parameters including central tendency (e.g. means) or other basic estimates (e.g. regression coefficient) AND variation (e.g. standard deviation) or associated estimates of uncertainty (e.g. confidence intervals)
☐	☒	For null hypothesis testing, the test statistic (e.g. *F*, *t*, *r*) with confidence intervals, effect sizes, degrees of freedom and *P* value noted<br>*Give P values as exact values whenever suitable.*
☐	☒	For Bayesian analysis, information on the choice of priors and Markov chain Monte Carlo settings
☒	☐	For hierarchical and complex designs, identification of the appropriate level for tests and full reporting of outcomes
☒	☐	Estimates of effect sizes (e.g. Cohen's *d*, Pearson's *r*), indicating how they were calculated

Our web collection on statistics for biologists contains articles on many of the points above.

Software and code

Policy information about availability of computer code

Data collection	Stimulus delivery and recordings of force data were controlled by Python.
Data analysis	FMRI data preprocessing and analysis was performed using SPM12 on MATLAB R2019b. The flat representation of the human cerebellum (cerebellar flatmap) provided with the SUIT toolbox (https://www.diedrichsenlab.org/imaging/suit.htm) was used to visualize the group average imaging data. The Wake Forest University Pickatlas toolbox was used for definition of Regions of Interest (ROIs).<br><br>Python 3.11.3 (libraries NumPy, SciPy) was used to preprocess the behavioral data. R (version 4.2.2, 2022-10-31) and JASP 0.16.4 were used for statistical analysis of the behavioral data.

For manuscripts utilizing custom algorithms or software that are central to the research but not yet described in published literature, software must be made available to editors and reviewers. We strongly encourage code deposition in a community repository (e.g. GitHub). See the Nature Portfolio guidelines for submitting code & software for further information.

Data

Policy information about availability of data

All manuscripts must include a data availability statement. This statement should provide the following information, where applicable:

- Accession codes, unique identifiers, or web links for publicly available datasets
- A description of any restrictions on data availability
- For clinical datasets or third party data, please ensure that the statement adheres to our policy

We do not have ethics approval to make the data publicly available.

Research involving human participants, their data, or biological material

Policy information about studies with human participants or human data. See also policy information about sex, gender (identity/presentation), and sexual orientation and race, ethnicity and racism.

Reporting on sex and gender	We were not interested in the effects of sex or gender, so no analyses were planned a priori based on sex or gender. We aimed to recruit equal numbers of male and female participants. Twelve (12) females and twelve (12) males participated in the experiment. These demographics are reported in the manuscript.
Reporting on race, ethnicity, or other socially relevant groupings	We do not refer to race, ethnicity or other socially relevant groupings in our manuscript.
Population characteristics	The experiment was conducted with a total of 24 participants: 12 women, 12 men; 22 right-handed, 2 ambidextrous; 19-36 years old. Participants self-reported having no current or history of psychological or neurological conditions, as well as the use of any psychoactive drugs or medication to treat such conditions.
Recruitment	The sample was recruited via online adverts posted on Facebook as well as Accindi (accindi.se). Participants were not eligible to participate if they had previously taken part in our previous experiments. We do not expect any selection biases to affect the data.
Ethics oversight	The Ethics Review Authority approved the study (project: #2016/445-31/2, amendment: #2018:1397-32).

Note that full information on the approval of the study protocol must also be provided in the manuscript.

Field-specific reporting

Please select the one below that is the best fit for your research. If you are not sure, read the appropriate sections before making your selection.

☒ Life sciences ☐ Behavioural & social sciences ☐ Ecological, evolutionary & environmental sciences

For a reference copy of the document with all sections, see nature.com/documents/nr-reporting-summary-flat.pdf

Life sciences study design

All studies must disclose on these points even when the disclosure is negative.

Sample size	We aimed for a sample size of 30 healthy participants based on our previous studies (Kilteni and Ehrsson, 2020, 2023).
Data exclusions	Due to a major scanner failure, one participant was not scanned at all. Five participants were further excluded: one for exiting the scanner in the middle of the fMRI runs, one for reporting extreme sleepiness during some of the fMRI runs, and three for technical problems with the setup or with registering their responses. Consequently, the behavioral and fMRI analyses included data from a total of 24 participants (12 women, 12 men; 22 right-handed, 2 ambidextrous; 19-36 years old).
Replication	Our study was the first attempt to test the effect of temporal recalibration for self-touch on brain activity. Our behavioral results replicated those of Kilteni et al. (2019, eLife). Our fMRI results from the baseline runs also replicated our previous fMRI results by Kilteni and Ehrsson (2020, Journal of Neuroscience) and Kilteni and Ehrsson (2023, Journal of Neuroscience).
Randomization	This was a within-subjects study. The order of the two sessions was randomized across participants.
Blinding	Participants were blind to the experimental manipulation (baseline or adaptation runs). The experimenters were not blind.

Reporting for specific materials, systems and methods

We require information from authors about some types of materials, experimental systems and methods used in many studies. Here, indicate whether each material, system or method listed is relevant to your study. If you are not sure if a list item applies to your research, read the appropriate section before selecting a response.

Materials & experimental systems

n/a	Involved in the study
☒	☐ Antibodies
☒	☐ Eukaryotic cell lines
☒	☐ Palaeontology and archaeology
☒	☐ Animals and other organisms
☒	☐ Clinical data
☒	☐ Dual use research of concern
☒	☐ Plants

Methods

n/a	Involved in the study
☒	☐ ChIP-seq
☒	☐ Flow cytometry
☐	☒ MRI-based neuroimaging

Plants

Seed stocks

Report on the source of all seed stocks or other plant material used. If applicable, state the seed stock centre and catalogue number. If plant specimens were collected from the field, describe the collection location, date and sampling procedures.

Novel plant genotypes

Describe the methods by which all novel plant genotypes were produced. This includes those generated by transgenic approaches, gene editing, chemical/radiation-based mutagenesis and hybridization. For transgenic lines, describe the transformation method, the number of independent lines analyzed and the generation upon which experiments were performed. For gene-edited lines, describe the editor used, the endogenous sequence targeted for editing, the targeting guide RNA sequence (if applicable) and how the editor was applied.

Authentication

Describe any authentication procedures for each seed stock used or novel genotype generated. Describe any experiments used to assess the effect of a mutation and, where applicable, how potential secondary effects (e.g. second site T-DNA insertions, mosiacism, off-target gene editing) were examined.

Magnetic resonance imaging

Experimental design

Design type

Event-related

Design specifications

Each session had three identical fMRI runs – the early, middle, and late runs – each consisting of 235 trials, on average, and including trials of both nondelayed and delayed self-generated touches.

Behavioral performance measures

To assess whether the participants pressed with forces of similar magnitude during the six fMRI and the six psychophysical runs, and thus rule out that any perceptual or neural effects are driven by differences in the produced or received forces rather than the injected delay, for each trial, we extracted the peak amplitudes of the test and active taps (defined as the peak force recording of each force sensor within the trial). We performed repeated measures ANOVA to test for differences between the runs.

Acquisition

Imaging type(s)

Functional and structural imaging

Field strength

3T

Sequence & imaging parameters

fMRI acquisition was performed using a General Electric 3T scanner (GE750 3T) equipped with an 8-channel head coil. Gradient echo T2*-weighted EPI sequences with BOLD contrast were used as an index of brain activity.

A functional image volume was composed of 42 slices (repetition time: 2000 ms; echo time: 30 ms; flip angle: 80 degrees; slice thickness: 3 mm; slice spacing: 3.5 mm; matrix size: 76 × 76; in-plane voxel resolution: 3 mm). A total of 155 functional volumes were collected for each participant during each run, resulting in a total of 930 functional volumes (155 volumes x 6 runs).

For the anatomical localization of activations, a high-resolution structural image containing 180 slices was acquired for each participant before the acquisition of the functional volumes (repetition time: 6.404 ms; echo time: 2.808 ms; flip angle: 12°; slice thickness: 1 mm; slice spacing: 1 mm; matrix size: 256 × 256; voxel size: 1 mm × 1 mm × 1 mm).

Area of acquisition

Whole brain scan

Diffusion MRI ☐ Used ☒ Not used

Preprocessing

Preprocessing software	A standard preprocessing pipeline was used, including realignment, unwarping and slice-time correction using Statistical Parametric Mapping 12 (SPM12; Welcome Department of Cognitive Neurology, London, UK, http://www.fil.ion.ucl.ac.uk/spm) software. After segmentation and normalization, the images were spatially smoothed using an 8-mm FWHM Gaussian kernel.
Normalization	We simultaneously segmented the images into gray matter, white matter and cerebrospinal fluid and normalized them into standard MNI space (Montreal Neurological Institute, Canada). The structural images were also simultaneously segmented (into gray and white matter and cerebrospinal fluid) and normalized to MNI space.
Normalization template	MNI
Noise and artifact removal	The six head motion parameters and any outlier volumes (see below) were included as regressors of no interest.
Volume censoring	Outlier volumes were detected using the Artifact Detection Tools, employing the option for liberal thresholds (global-signal threshold of z = 9 and subject-motion threshold of 2 mm).

Statistical modeling & inference

Model type and settings	BOLD signal responses were modeled by fitting voxelwise GLMs to the data of each fMRI run. For all six runs, the main regressor of interest was deviant touches (i.e., 12% delayed self-generated touches in the early, middle, and late runs of the baseline session, and 12% nondelayed touches in the early, middle, and late runs of the adaptation session), while the repeated standard touches (i.e., 88% nondelayed self-generated touches in the early, middle, and late runs of the baseline session, and 88% delayed touches in the early, middle, and late runs of the adaptation session) were modelled as the "implicit" baseline. The onset of the trials was defined as the time when the magnitude of the test tap peaked, their duration was set to zero, and they were convolved with the canonical hemodynamic response function of SPM12. Any trials in which the participants did not tap the sensor with their right index finger after the auditory cue, tapped too lightly to trigger the touch on the left index finger (active tap < 0.4 N), tapped more than once, or tapped before the auditory GO cue were excluded from the regressor of interest and implicit baseline and modeled as four (4) individual regressors of no interest. According to these criteria, we excluded 805 trials out of 34682 fMRI trials (2.3%) from the main regressor of interest and the implicit baseline, leading to a total of 33877 valid trials. The number of excluded trials varied per participant (mean +/- standard deviation = 6 +/- 10 trials per session). In addition, the six motion parameters and any outlier volumes were included as regressors of no interest. To account for the potential influence of small variations in the magnitude of the self-generated force of active taps on the BOLD signal, we also included the magnitude of the active tap on each trial as a parametric modulator. Finally, the first-level analysis was restricted to gray matter voxels using a binary (threshold of 0.2) and smoothed mask (8-mm FWHM Gaussian kernel) of gray matter, which was based on the individual's segmented structural image (gray matter).
Effect(s) tested	Six contrasts against the implicit baseline were created for all the deviant self-generated touches. These contrasts were inserted in a 2 x 3 full factorial design with two within-subjects factors: session (adaptation or baseline) and run (early, middle, late).

Specify type of analysis: ☐ Whole brain ☐ ROI-based ☒ Both

Anatomical location(s)	We performed small-volume corrections within regions of interest (ROIs). The somatosensory ROIs included the right primary somatosensory cortex, defined as a spherical region of 10-mm radius, centered at a peak detected in our previous study (MNI coordinates: x = 50, y = -20, z = 60) using the same scanner, same equipment, and same tactile stimulation (2 N) applied to the same finger (left index finger). The right secondary somatosensory cortex was defined using the Anatomy Toolbox 99 by selecting the Brodmann area OP1(SII). Two ROIs included the hemispheres of the left cerebellar lobules IV-VI and VIII, and one ROI included the anterior cingulate cortex, all defined with the Wake Forest University Pickatlas toolbox. In addition to these ROIs, we also report analyses at the whole-brain level.
Statistic type for inference (See Eklund et al. 2016)	Voxel-wise
Correction	For each peak activation, the coordinates in MNI space, the z value and the p value are reported. We denote that a peak survived a threshold of p < 0.05 after correction for multiple comparisons at the whole-brain or small-volume level by adding the term "FWEWB" and "FWESV" after the p value, respectively.

Models & analysis

n/a	Involved in the study
☒	☐ Functional and/or effective connectivity
☒	☐ Graph analysis
☒	☐ Multivariate modeling or predictive analysis
